# Enabling Next-Generation Structural Science with Cloud and Edge Computing

**DOI:** 10.1063/4.0000938

**Published:** 2025-10-27

**Authors:** Max Burian, Ludmilla Leroy, Fabian Eisenstein, Pascal Hofer

**Affiliations:** DECTRIS Ltd., Baden, AG, Switzerland

## Abstract

Modern crystallographic research is experiencing unprecedented increases in data rates, driven by advanced detector technologies, faster beamlines, higher brilliance, and increasingly automated experiments. Efficiently managing and processing these large datasets presents significant challenges, including storage scalability, rapid data accessibility, and effective implementation of FAIR (Findable, Accessible, Interoperable, and Reusable) data principles.

Here, we discuss scientific strategies and technological innovations developed at DECTRIS to address these challenges. Leveraging cloud-based solutions, we demonstrate how researchers can seamlessly integrate large-volume crystallographic data processing workflows for Protein Crystallography, Serial Crystallography and Scanning Diffraction Experiments, enabling rapid data processing, analysis and visualization. We focus on technological advantages of cloud computing, such as software containerization and object store based data access, by showcasing specific use cases from synchrotrons such as MaxIV and SLS2.0.

Additionally, we explore the role of edge computing in crystallographic experiments, detailing how intelligent data handling at the detector edge reduces bandwidth demands, accelerates real-time decision-making and helps reduce data amounts through e.g. vetoing approaches.

We illustrate the integration of these cloud and edge approaches through collaborative studies conducted at various synchrotron and electron microscopy facilities, emphasizing the scientific benefits of scalable data processing. Ultimately, these strategies empower structural scientists to more effectively harness high data rates, enhancing the speed, reproducibility, and overall impact of crystallographic research.

